# The Surgical Disorders of the Urinary Organs

**Published:** 1887-09

**Authors:** 


*The Surgical Disorders of the Urinary Organs.* 3rd edition. By REGINALD HARRISON, F.R.C.S. Pp. 583. London: J. & A. CHURCHILL. 1887.

The third edition of Mr. Harrison's valuable and popular work has been entirely re-written, and has been enriched by the addition of much new matter. In the department of surgery covered by these lectures the author holds a very high reputation, and we may justly look upon any work from his pen as representing the highest level of excellence to which the surgical treatment of Diseases of the Urinary Organs has attained. But the work before us is more than this. It is personal in the best sense of the word; not only as embodying the teachings of an extensive experience in the ordinary paths of practice, but as breaking new ground, and devising original and ingenious plans of treatment. Mr. Harrison is also a diligent student of current literature, and on every page gives happy illustrations, from the most divergent sources, of some theory he desires to illustrate or some principle he wishes to enforce.

The style of writing is most captivating. It is clear, fresh, unaffected, and always to the point. We know of no recent surgical work whose perusal is likely to give the reader more profit and pleasure than this one.

